# Immune-related core gene identification in irritable bowel syndrome and prediction of regulatory traditional Chinese medicines

**DOI:** 10.1097/MD.0000000000048430

**Published:** 2026-05-08

**Authors:** Wei Bai, Zixing Qian, Yang Yang, Guodong Huang, Xianjun Rao, Hao Li, Tingting Zhou, Wei Wei

**Affiliations:** a Beijing University of Chinese Medicine, Beijing, China; b Wang Jing Hospital of China Academy of Chinese Medical Sciences, Beijing, China; c Guangzhou University of Chinese Medicine, Guangzhou, China.

**Keywords:** bioinformatics, immune-related genes, irritable bowel syndrome, machine learning, traditional Chinese medicine

## Abstract

Immune dysregulation is increasingly recognized as an important contributor to the pathophysiology of irritable bowel syndrome (IBS). This study aimed to identify immune-related core genes in IBS and predict potential regulatory traditional Chinese medicines (TCMs). Two IBS-related Gene Expression Omnibus datasets were integrated and analyzed to identify differentially expressed genes (DEGs). Functional enrichment, immune cell infiltration profiling, and intersection with ImmPort immune-related genes were performed. Core genes were selected through 3 machine-learning algorithms, and potential TCMs were predicted using the Coremine Medical database. A total of 95 DEGs were identified, including 56 upregulated and 39 downregulated genes. Enrichment analyses indicated involvement in muscle system processes, membrane-associated structures, and peptidase inhibitor activity, with significant enrichment in the neuroactive ligand–receptor interaction pathway. Immune infiltration analysis showed increased M2 macrophages, resting natural killer cells, resting dendritic cells, and activated mast cells in IBS samples. Seven immune-related DEGs were obtained, among which *LEFTY1*, *SLPI*, and *INSL5* were consistently recognized as core genes across all machine-learning approaches. These genes exhibited distinct immune regulatory relevance. Five TCMs: Drynaria fortunei, Crataegus pinnatifida, Houttuynia cordata, Poria cocos, and Bubalus bubalis horn were predicted as potential therapeutic agents targeting the core genes. *LEFTY1*, *SLPI*, and *INSL5* represent key immune-related genes in IBS and may contribute to its immune regulatory mechanisms. The predicted TCMs provide potential candidates for further validation in IBS management.

## 1. Introduction

Irritable bowel syndrome (IBS) is a common functional gastrointestinal disorder characterized by recurrent abdominal pain accompanied by alterations in bowel habits, in the absence of structural or biochemical abnormalities.^[1]^ As a chronic and relapsing condition, IBS affects 7% to 21% of individuals worldwide, imposing a substantial impact on quality of life and healthcare resources.^[2,3]^ According to the Rome IV criteria, IBS is classified into constipation-predominant (IBS-C), diarrhea-predominant (IBS-D), mixed (IBS-M), and unclassified (IBS-U) subtypes.^[4]^ Evidence from Western populations indicates a higher prevalence of IBS among women than men, potentially related to estrogen-mediated modulation of visceral sensitivity or sex-specific immune alterations.^[5]^ The recurrent nature of IBS symptoms contributes to significant impairment in daily functioning, reduced work productivity, and considerable economic burden.^[6,7]^

Current investigations into IBS pathophysiology suggest that the disorder results from multiple interacting mechanisms, including dysfunction of the gut–brain axis, immune dysregulation, alterations in gut microbiota, and genetic and epigenetic influences.^[8]^ Notably, aberrant immune activity has been increasingly documented in IBS. Studies have shown activation of the colonic mucosal immune system in IBS patients, characterized by infiltration of immune cells and increased production of inflammatory cytokines.^[9]^ Local immune dysregulation within the intestinal mucosa is therefore considered a key contributor to symptom generation and disease progression in IBS. Traditional Chinese medicine (TCM) has shown therapeutic advantages in IBS management. Evidence indicates that herbal medicines can effectively alleviate abdominal pain, constipation, and diarrhea while exhibiting a relatively low risk of adverse effects.^[10,11]^ Nevertheless, the molecular mechanisms underlying TCM-mediated immunoregulation in IBS remain insufficiently elucidated.

Given the central role of immune imbalance in IBS and the potential immunomodulatory properties of TCM, identifying immune-related biomarkers may facilitate the discovery of novel therapeutic targets. Therefore, this study aims to screen immune-related core genes associated with IBS through integrated bioinformatics analyses and to predict regulatory TCM candidates, providing a theoretical foundation for further mechanistic research and TCM-based interventions.

## 2. Methods

### 2.1. Data acquisition and integration

This study was approved by the Ethics Committee of Wang Jing Hospital (2025-WJH-1012kf). Two IBS-related gene expression datasets, GSE14841 and GSE36701, were obtained from the Gene Expression Omnibus (GEO) database (https://www.ncbi.nlm.nih.gov/geo/). GSE14841 included 9 IBS samples, while GSE36701 contained 164 samples (77 healthy controls and 87 IBS cases). The 2 datasets were merged using R software after probe annotation and normalization. Because the datasets originated from different experimental batches, batch effects were removed using the sva package. The corrected expression matrix was used for all subsequent analyses.

### 2.2. Differential expression analysis

Differentially expressed genes (DEGs) were identified using the limma package. Genes with |log_2_ fold change| > 1 and adjusted *P* < .05 were considered significant DEGs. Volcano plots were generated to visualize the distribution of upregulated and downregulated genes.

### 2.3. Functional enrichment analysis

Functional annotation of DEGs was performed using the clusterProfiler package for Gene Ontology (GO) and Kyoto Encyclopedia of Genes and Genomes (KEGG) enrichment analyses. GO enrichment was conducted across the biological process, cellular component, and molecular function categories. KEGG pathway analysis was performed with a threshold of *P* < .05. Bubble plots were generated to display significantly enriched biological terms and pathways.

### 2.4. Immune cell infiltration analysis

To characterize immune infiltration patterns in IBS, the Cell-type Identification by Estimating Relative Subsets of RNA Transcripts algorithm was applied to the normalized expression matrix to estimate the relative abundance of 22 immune cell types. The number of permutations was set to 1000 to ensure analytical robustness. Violin plots were used to compare immune cell proportions between the IBS and control groups.

### 2.5. Immune cell correlation analysis

Correlation patterns among immune cell subsets were assessed using Pearson correlation coefficients, computed with the corrplot package. A correlation heatmap was generated to visualize intercellular associations and potential immune regulatory interactions.

### 2.6. Identification of immune-related DEGs

Immune-related genes were retrieved from the ImmPort database (https://www.immport.org/). Immune-related DEGs were obtained by intersecting ImmPort immune gene lists with previously identified DEGs, yielding candidate genes associated with immune regulation in IBS.

### 2.7. Core gene selection using machine-learning algorithms

To identify the most informative immune-related genes, 3 machine-learning approaches were applied to the candidate immune-related DEGs: least absolute shrinkage and selection operator regression, performed using the glmnet package with tenfold cross-validation to determine the optimal lambda. Genes with nonzero coefficients were retained; support vector machine–recursive feature elimination: Executed with the RFE function to iteratively remove less informative features and identify genes with the highest classification accuracy; and Random Forest: Constructed using the random Forest package. Gene importance was ranked based on the MeanDecreaseGini index. Genes identified by all 3 algorithms were considered core IBS-related immune genes.

### 2.8. Prediction of potential traditional Chinese medicines

Core genes were uploaded to the Coremine Medical database to identify associated TCMs. Only TCMs with *P* < .05 and strong association scores were retained as candidate agents. The predicted herbs were then cross-referenced with the 2020 edition of the Pharmacopoeia of the People’s Republic of China to ensure inclusion of clinically recognized TCMs.

## 3. Results

### 3.1. Identification of differentially expressed genes

**Figure 1.**
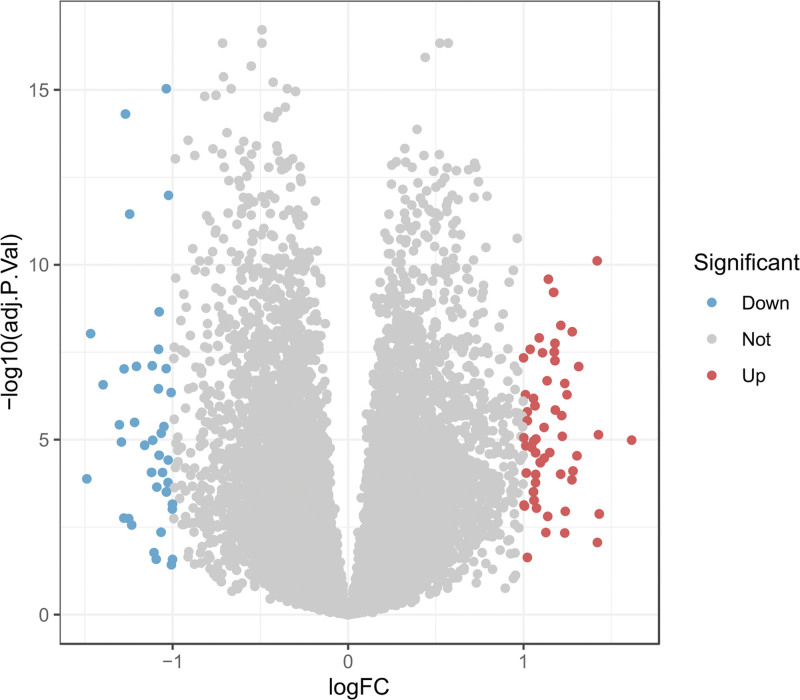
Volcano plot of differential expression analysis.

A total of 95 DEGs were identified from the integrated dataset using the limma package, including 56 upregulated and 39 downregulated genes in IBS samples compared with controls. The volcano plot demonstrated distinct segregation of significantly upregulated genes (red), downregulated genes (blue), and nonsignificant genes (gray) (Fig. 1).

### 3.2. Functional enrichment analysis

**Figure 2.**
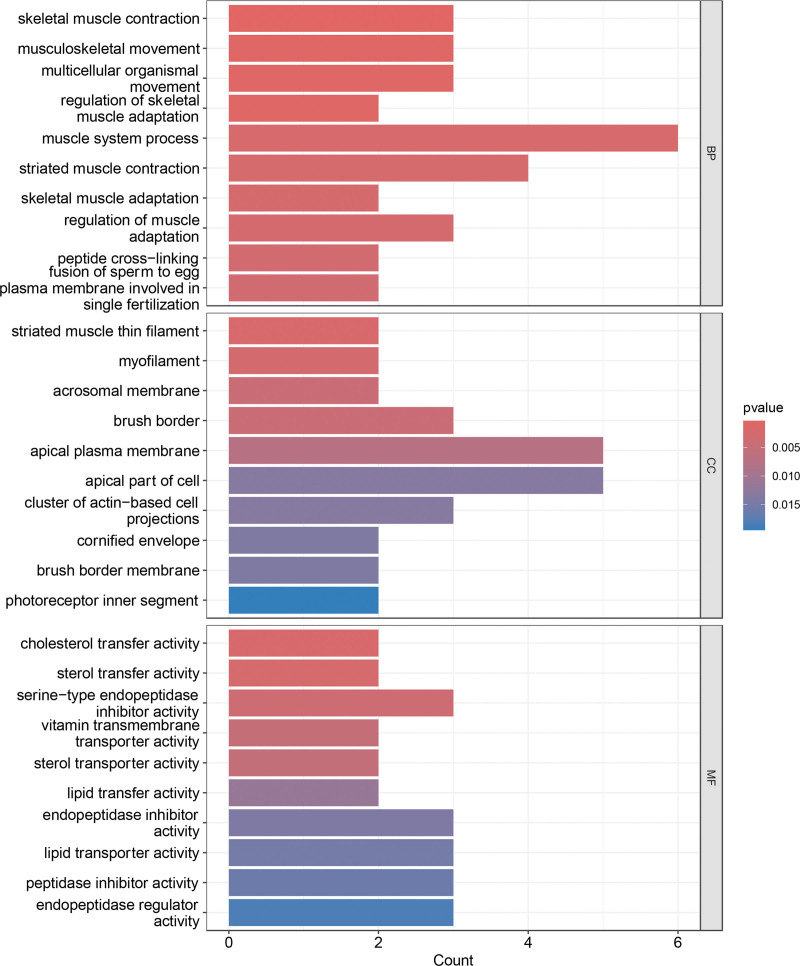
GO analysis. GO = Gene Ontology.

**Figure 3.**
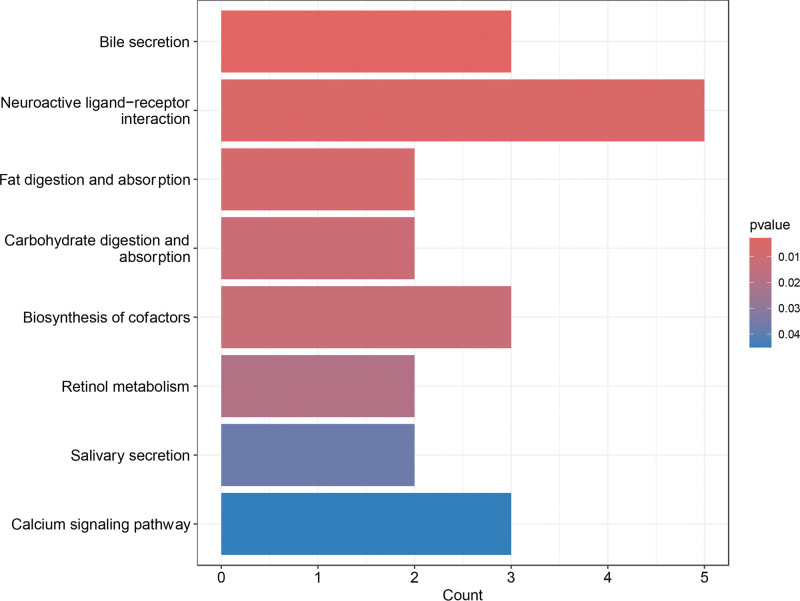
KEGG analysis. KEGG = Kyoto Encyclopedia of Genes and Genomes.

GO enrichment analysis revealed significant involvement of DEGs in multiple biological processes, notably muscle system processes, striated muscle contraction, and skeletal muscle contraction. In the cellular component category, DEGs were enriched in the apical plasma membrane, apical cell region, and brush border, indicating predominant localization to membrane-associated structures. In the molecular function category, DEGs were enriched in serine-type endopeptidase inhibitor activity, endopeptidase inhibitor activity, and lipid transporter activity, suggesting potential roles in enzyme regulation and lipid metabolism (Fig. 2). KEGG pathway analysis demonstrated significant enrichment in neuroactive ligand–receptor interaction, bile secretion, and cofactor biosynthesis pathways, indicating potential relevance to gastrointestinal motility and mucosal signaling processes (Fig. 3).

### 3.3. Immune cell infiltration analysis

**Figure 4.**
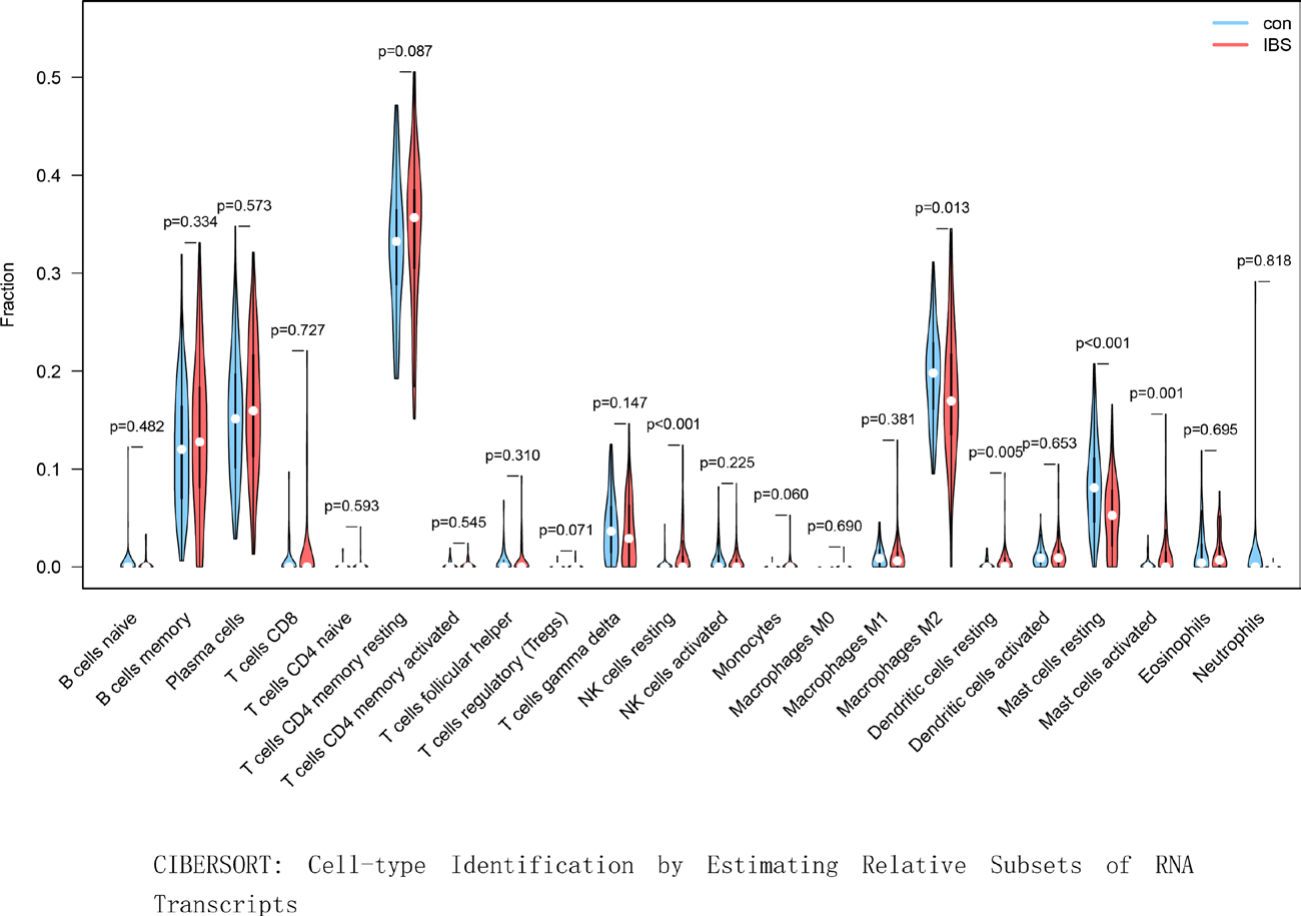
CIBERSORT analysis vioplot. CIBERSORT = Cell-type Identification by estimating relative subsets of RNA transcripts.

Cell-type Identification by Estimating Relative Subsets of RNA Transcripts analysis revealed significant alterations in immune cell composition in IBS samples. M2 macrophages (*P* = .013), resting natural killer (NK) cells (*P* < .001), resting dendritic cells (*P* = .005), and activated mast cells (*P* = .001) were markedly increased in IBS compared with controls. Conversely, resting mast cells were significantly more abundant in the control group (*P* < .001) (Fig. 4). These findings suggest that immune cell activation and polarization may contribute to IBS-related immune dysregulation.

### 3.4. Correlation among immune cell subsets

**Figure 5.**
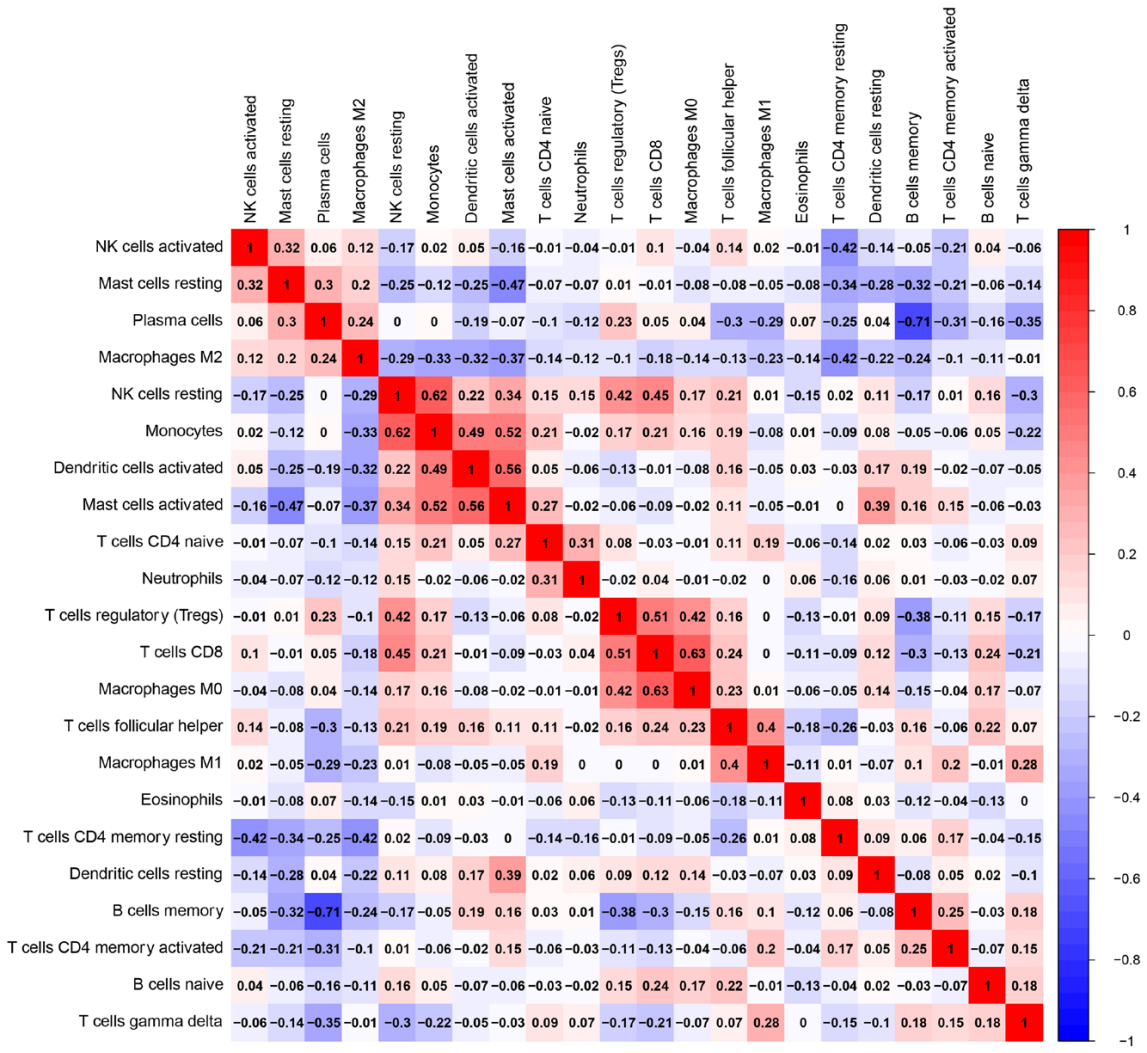
Correlation heatmap of immune cells.

Correlation heatmap analysis demonstrated distinct patterns of immune cell interactions (Fig. 5). Monocytes showed a strong positive correlation with resting NK cells (*R* = 0.62), whereas M0 macrophages were positively correlated with CD8^+^ T cells (*R* = 0.63). Memory B cells exhibited a negative correlation with plasma cells (*r* = –0.71). These associations indicate potential cooperative or opposing immune regulatory patterns within the IBS immune microenvironment.

### 3.5. Identification of immune-related differentially expressed genes

**Figure 6.**
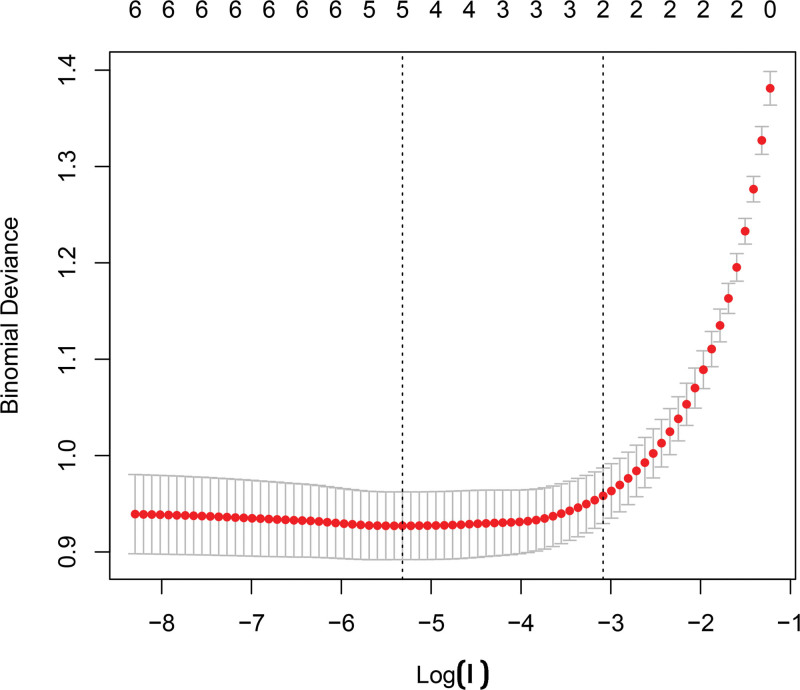
LASSO analysis. LASSO = least absolute shrinkage and selection operator.

Intersection of DEGs with immune-related genes from the ImmPort database identified 7 immune-related DEGs: *PTH2*, *LEFTY1*, *SLPI*, PI3, *AVPR1B*, *GNRH2*, and *INSL5*. These genes represent IBS-related immune regulatory candidates with potential functional relevance (Fig. 6).

### 3.6. Selection of core immune-related genes

**Figure 7.**
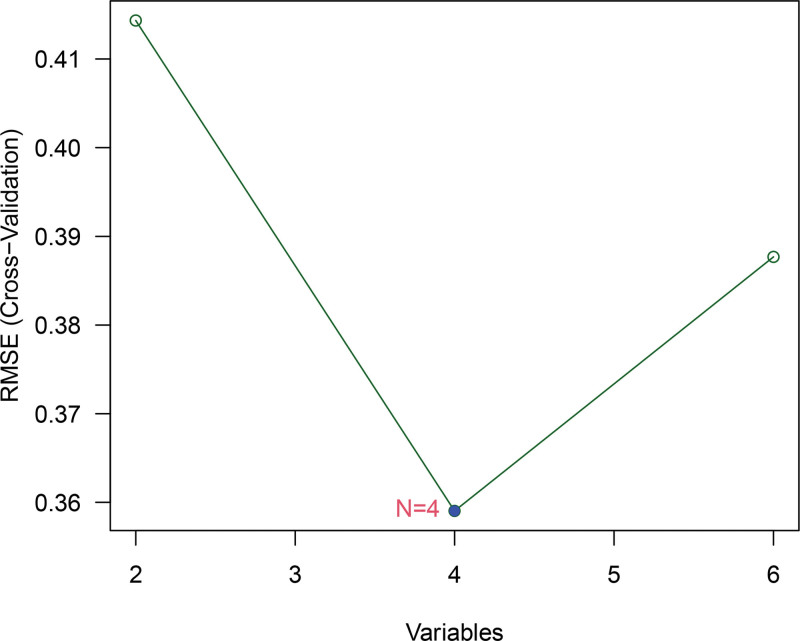
SVM-RFE analysis. SVM-RFE = Support Vector Machine–Recursive Feature Elimination.

**Figure 8.**
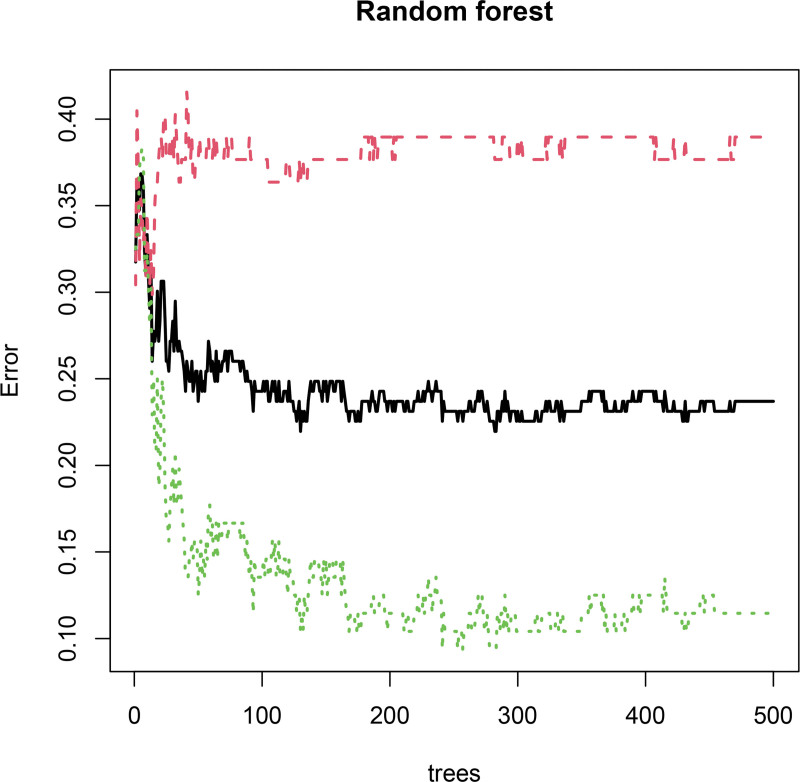
Random forest analysis.

**Figure 9.**
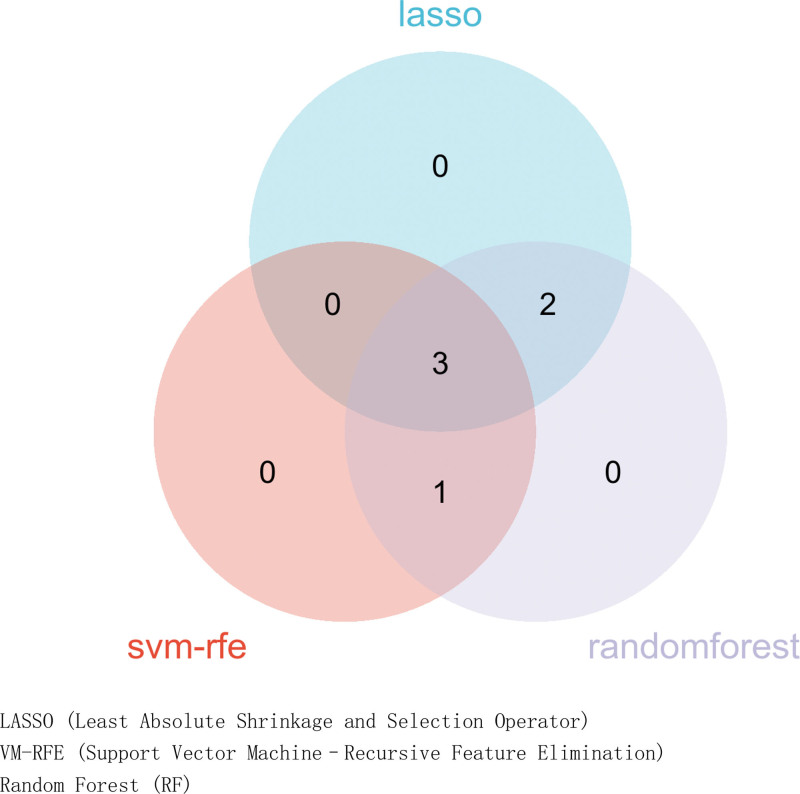
Venn diagram of LASSO, SVM-RFE, and random forest analysis. LASSO = least absolute shrinkage and selection operator, LASSO = least absolute shrinkage and selection operator.

Three machine-learning algorithms were applied to the 7 immune-related DEGs. Least absolute shrinkage and selection operator regression identified 5 genes: *LEFTY1*, *SLPI*, *AVPR1B*, *GNRH2*, and *INSL5* (Fig. 7). Support vector machine–recursive feature elimination selected 4 genes: *LEFTY1*, *INSL5*, *SLPI*, and PI3 (Fig. 8). Random forest analysis selected 6 genes: *LEFTY1*, *INSL5*, *SLPI*, PI3, *GNRH2*, and *AVPR1B* (Fig. 9). Venn diagram integration of the 3 algorithm outputs identified 3 consistently selected core genes: *LEFTY1*, *SLPI*, and *INSL5*. These genes may serve as key immune-related molecular markers in the pathophysiology of IBS.

### 3.7. Prediction of potential traditional Chinese medicines

Core genes were input into the Coremine Medical database to identify associated TCMs. Three herbs were linked to *INSL5,* and 5 herbs were linked to *SLPI*, whereas no significant TCM associations were identified for *LEFTY1*. Following cross-referencing with the 2020 edition of the Pharmacopoeia of the People’s Republic of China, 5 TCMs were retained as candidate regulatory agents: Drynaria fortunei, Crataegus pinnatifida, Houttuynia cordata, Poria cocos, and Bubalus bubalis horn.

## 4. Discussion

This study integrated bioinformatics and machine-learning approaches to identify immune-related molecular signatures associated with IBS. By merging 2 independent GEO datasets and applying batch-effect correction, 95 DEG were identified. Functional enrichment analyses revealed that these genes were involved in pathways related to immune regulation, inflammatory responses, and gastrointestinal physiological processes. Intersection with immune gene sets from the ImmPort database yielded 7 immune-related DEGs, and subsequent machine-learning analyses consistently identified *LEFTY1*, *SLPI*, and *INSL5* as core immune-related genes. These findings suggest that dysregulation of specific immune pathways may contribute to IBS pathophysiology.

*LEFTY1*, a member of the transforming growth factor-β (TGF-β) superfamily, has been implicated in immune modulation and anti-inflammatory signaling. Prior studies indicate that *LEFTY1* can attenuate inflammation by suppressing the NF-κB pathway, a central mediator of immune and inflammatory responses.^[12,13]^ Although its role in IBS has not been directly characterized, the established regulatory effects of *LEFTY1* suggest its potential involvement in mucosal immune homeostasis. *SLPI* is an anti-inflammatory protein known to inhibit the release of pro-inflammatory cytokines and regulate innate immune responses.^[14,15]^ Although evidence linking *SLPI* to IBS is limited, its regulatory function in immune activation suggests that dysregulated *SLPI* expression may contribute to the immune imbalance observed in IBS patients. *INSL5* is a peptide hormone predominantly expressed in the distal colon and rectum, and its receptor, relaxin family peptide receptor 4, is widely distributed in gastrointestinal and neural tissues.^[16]^ Emerging evidence suggests that *INSL5*– relaxin family peptide receptor 4 signaling may influence immune processes and intestinal homeostasis. Its high expression in the distal gut implicates *INSL5* as a potential mediator of local immune regulation in IBS.

Immune cell infiltration analysis further supported the involvement of altered immunity in IBS. Increased proportions of M2 macrophages, resting NK cells, resting dendritic cells, and activated mast cells were observed in IBS samples. M2 macrophages possess anti-inflammatory properties and may reflect compensatory responses to chronic mucosal irritation.^[17]^ NK cells have diverse immunomodulatory roles and may influence intestinal homeostasis through interactions within the gut-associated lymphoid tissue.^[18]^ Mast cell activation, previously linked to visceral hypersensitivity and abdominal pain, is a well-recognized feature of IBS pathogenesis.^[19,20]^ The identified immune cell alterations provide insights into the complex immunological microenvironment characteristic of IBS.

Prediction of potential TCMs based on core genes identified 5 candidate herbs. Existing evidence indicates that components from Drynaria fortunei, particularly diosgenin saponins, may regulate gastrointestinal peptides such as vasoactive intestinal peptide, 5-hydroxytryptamine, and substance P, thereby influencing inflammation-related and immune regulatory pathways. Poria cocos has been shown to modulate gut microbiota, reinforce mucosal barrier integrity, and attenuate intestinal inflammation, particularly in IBS-D.^[21]^ Crataegus pinnatifida is traditionally used to address gastrointestinal dysfunction and has been reported to relieve symptoms of IBS-D through regulation of digestive and motility-related pathways. However, evidence for the therapeutic relevance of Houttuynia cordata and Bubalus bubalis horn in IBS remains limited, warranting further investigation.^[22,23]^

Overall, this study provides new insights into the immune-related molecular landscape of IBS and highlights potential TCM candidates for therapeutic modulation. The integration of bioinformatics and machine-learning algorithms enhances the reliability of core gene identification. Nevertheless, this study has certain limitations. The analyses were based on publicly available transcriptomic datasets, and validation using clinical samples or experimental models was not performed. Additionally, the predicted TCM gene associations were derived from database-based correlation analyses, necessitating further pharmacological and mechanistic studies. Future work should focus on experimental validation of the identified core genes, functional characterization of their roles in intestinal immunity, and in vivo assessment of the predicted TCMs. Such investigations may contribute to the development of targeted immunomodulatory strategies and support the modernization of TCM-based IBS therapies.

Several limitations of this study should be acknowledged. First, although 2 GEO datasets were integrated and batch effects were corrected using the sva package, an independent external GEO dataset was not available for validation. Therefore, the identified core genes should be regarded as exploratory findings rather than definitive biomarkers. Future studies using independent cohorts and experimental validation are warranted to confirm the robustness and clinical relevance of these results. In addition, data heterogeneity may have influenced the stability of the results. The included datasets differed in sample size, experimental platforms, and potential clinical characteristics of the enrolled subjects, which may introduce variability despite batch-effect correction. Such heterogeneity could affect the reproducibility of differential gene expression and immune infiltration patterns, and this should be considered when interpreting the findings.

## 5. Conclusions

This study identified *LEFTY1*, *SLPI*, and *INSL5* as immune-related core genes associated with IBS through integrated bioinformatics and machine-learning algorithms. Altered immune cell infiltration patterns further support the involvement of immune dysregulation in IBS. In addition, several TCMs were predicted as potential candidate agents with hypothetical associations to these core genes. These findings provide exploratory insights and a theoretical basis for future experimental validation and mechanistic investigations.

## Author contributions

**Conceptualization:** Wei Bai, Zixing Qian, Yang Yang, Guodong Huang, Hao Li, Tingting Zhou, Wei Wei.

**Data curation:** Wei Bai, Zixing Qian, Yang Yang, Guodong Huang, Hao Li, Tingting Zhou, Wei Wei.

**Formal analysis:** Wei Bai, Zixing Qian, Yang Yang, Guodong Huang, Xianjun Rao, Hao Li, Tingting Zhou, Wei Wei.

**Funding acquisition:** Wei Bai, Zixing Qian, Wei Wei.

**Investigation:** Wei Bai, Wei Wei.

**Writing – original draft:** Wei Bai, Xianjun Rao, Tingting Zhou, Wei Wei.

**Writing – review & editing:** Wei Bai, Zixing Qian, Xianjun Rao, Hao Li, Wei Wei.

## References

[1] Drossman DA. Functional gastrointestinal disorders: history, pathophysiology, clinical features and Rome IV. Gastroenterology. 2016;150:S0016-5085(16)00223-7. 10.1053/j.gastro.2016.02.032 27144617

[2] Huang KY Wang FY Lv M Ma XX Tang XD Lv L. Irritable bowel syndrome: epidemiology, overlap disorders, pathophysiology and treatment. World J Gastroenterol. 2023;29:4120–35. 37475846 10.3748/wjg.v29.i26.4120 PMC10354571

[3] Lovell RM Ford AC. Global prevalence of and risk factors for irritable bowel syndrome: a meta-analysis. Clin Gastroenterol Hepatol. 2012;10:712–21.e4. 22426087 10.1016/j.cgh.2012.02.029

[4] Sebastián Domingo JJ. Irritable bowel syndrome. Med Clin (Barc). 2022;158:76–81. 34238582 10.1016/j.medcli.2021.04.029

[5] Meleine M Matricon J. Gender-related differences in irritable bowel syndrome: potential mechanisms of sex hormones. World J Gastroenterol. 2014;20:6725–43. 24944465 10.3748/wjg.v20.i22.6725 PMC4051914

[6] Sperber AD Bangdiwala SI Drossman DA. Worldwide prevalence and burden of functional gastrointestinal disorders, results of Rome foundation global study. Gastroenterology. 2021;160:99–114.e3. 32294476 10.1053/j.gastro.2020.04.014

[7] Konings B Balsiger LM Hreinsson JP. Global prevalence and gastrointestinal symptom burden of individuals with a history of cholecystectomy. Gut. 2025;75:65–71. Published 2025 Dec 5 40803749 10.1136/gutjnl-2024-334531

[8] Chong PP Chin VK Looi CY Wong WF Madhavan P Yong VC. The microbiome and irritable bowel syndrome - a review on the pathophysiology, current research and future therapy. Front Microbiol. 2019;10:1136. Published 2019 Jun 10 31244784 10.3389/fmicb.2019.01136 PMC6579922

[9] Sbidian E Chaimani A Garcia-Doval I. Systemic pharmacological treatments for chronic plaque psoriasis: a network meta-analysis. Cochrane Database Syst Rev. 2021;4:CD011535. Published 2021 Apr 19 33871055 10.1002/14651858.CD011535.pub4 PMC8408312

[10] Wu YB Dai YK Zhang L. Pharmacological treatments of Chinese herbal medicine for irritable bowel syndrome in adults: a network meta-analysis of randomized controlled trials. PLoS One. 2021;16:e0255665. Published 2021 Aug 6 34358263 10.1371/journal.pone.0255665 PMC8345858

[11] Zheng H Jin S Shen YL. Chinese herbal medicine for irritable bowel syndrome: a meta-analysis and trial sequential analysis of randomized controlled trials. Front Pharmacol. 2021;12:694741. Published 2021 Jul 27 34385918 10.3389/fphar.2021.694741 PMC8353248

[12] Zhang L Xu C Hu W Wu P Qin C Zhang J. Anti-inflammatory effects of Lefty-1 in renal tubulointerstitial inflammation via regulation of the NF-κB pathway. Int J Mol Med. 2018;41:1293–304. 29286065 10.3892/ijmm.2017.3327 PMC5819905

[13] Tak PP Firestein GS. NF-kappaB: a key role in inflammatory diseases. J Clin Invest. 2001;107:7–11. 11134171 10.1172/JCI11830 PMC198552

[14] Müller AM Jun E Conlon H Sadiq SA. Inhibition of *SLPI* ameliorates disease activity in experimental autoimmune encephalomyelitis. BMC Neurosci. 2012;13:30. Published 2012 Mar 21 22436018 10.1186/1471-2202-13-30 PMC3352067

[15] Zakrzewicz A Richter K Zakrzewicz D. *SLPI* inhibits ATP-mediated maturation of IL-1β in human monocytic leukocytes: a novel function of an old player. Front Immunol. 2019;10:664. Published 2019 Apr 4 31019507 10.3389/fimmu.2019.00664 PMC6458293

[16] Ang SY Evans BA Poole DP. *INSL5* activates multiple signalling pathways and regulates GLP-1 secretion in NCI-H716 cells. J Mol Endocrinol. 2018;60:213–24. 29535183 10.1530/JME-17-0152

[17] Han X Ding S Jiang H Liu G. Roles of macrophages in the development and treatment of gut inflammation. Front Cell Dev Biol. 2021;9:625423. Published 2021 Mar 2 33738283 10.3389/fcell.2021.625423 PMC7960654

[18] Chai WH Ma Y Li JJ Guo F Wu YZ Liu JW. Immune cell signatures and causal association with irritable bowel syndrome: a Mendelian randomization study. World J Clin Cases. 2024;12:3094–104. 38898868 10.12998/wjcc.v12.i17.3094 PMC11185378

[19] Uranga JA Martínez V Abalo R. Mast cell regulation and irritable bowel syndrome: effects of food components with potential nutraceutical use. Molecules. 2020;25:4314. Published 2020 Sep 20 32962285 10.3390/molecules25184314 PMC7570512

[20] Krammer L Sowa AS Lorentz A. Mast cells in irritable bowel syndrome: a systematic review. J Gastrointestin Liver Dis. 2019;28:463–72. Published 2019 Dec 9 31826052 10.15403/jgld-229

[21] Hao L Yu Z Sun J. Supplementation of Crataegi fructus alleviates functional dyspepsia and restores gut microbiota in mice. Front Nutr. 2024;11:1385159. Published 2024 Apr 2 38628273 10.3389/fnut.2024.1385159 PMC11018912

[22] Liu F Zhang X Ji Y. Total flavonoid extract from hawthorn (Crataegus pinnatifida) improves inflammatory cytokines-evoked epithelial barrier deficit. Med Sci Monit. 2020;26:e920170. Published 2020 Feb 17 32065826 10.12659/MSM.920170 PMC7041422

[23] Zhou X Li Y Yang Y. Regulatory effects of Poria cocos polysaccharides on gut microbiota and metabolites: evaluation of prebiotic potential. NPJ Sci Food. 2025;9:53. Published 2025 Apr 22 40263347 10.1038/s41538-025-00416-9 PMC12015419

